# EpEX/EpCAM and Oct4 or Klf4 alone are sufficient to generate induced pluripotent stem cells through STAT3 and HIF2α

**DOI:** 10.1038/srep41852

**Published:** 2017-02-03

**Authors:** I.-I. Kuan, Kang-Hao Liang, Yi-Ping Wang, Ting-Wen Kuo, Yaa-Jyuhn James Meir, Sareina Chiung-Yuan Wu, Shang-Chih Yang, Jean Lu, Han-Chung Wu

**Affiliations:** 1 Institute of Cellular and Organismic Biology, Academia Sinica, Taipei, Taiwan; 2 Department of Life Science, National Taiwan University, Taipei, Taiwan; 3 School of Dentistry, National Taiwan University, Taipei, Taiwan; 4 Department of Clinical Medicine, School of Medicine, Zhejang University, Zhejiang, China; 5 Institute of Molecular Medicine, College of Medicine, Chang Gung University, Tao-Yuan, Taiwan; 6 Genomics Research Center, Academia Sinica, Taipei, Taiwan

## Abstract

Epithelial cell adhesion molecule (EpCAM) was reported to be cleaved into extracellular domain of EpCAM (EpEX) and intracellular domain of EpCAM (EpICD). We previously reported that EpCAM serves as a potent stem cell marker which is highly and selectively expressed by undifferentiated rather than differentiated hESC. However, the functional role of EpCAM remains elusive. Here, we found that EpEX and EpCAM enhance the efficiency of OSKM reprogramming. Interestingly, Oct4 or Klf4 alone, but not Sox2, can successfully reprogram fibroblasts into iPSCs with EpEX and EpCAM. Moreover, EpEX and EpCAM trigger reprogramming via activation of STAT3, which leads to the nuclear-translocation of HIF2α. This study reveals the importance of a novel EpEX/EpCAM-STAT3-HIF2α signal in the reprogramming process, and uncovers a new means of triggering reprogramming by delivery of soluble and transmembrane proteins.

Embryonic stem cells (ESCs) are able to generate all three germ layers and differentiate into all cell types of the body, except placenta^1^. However, the application of ESCs in regenerative medicine was hampered due to immune-rejection and oncogenicity. Fortunately, the prospects for regenerative research were advanced by the groundbreaking discovery that induced pluripotent stem cells (iPSCs) can be established from somatic cells, such as fibroblasts^2^. The use of iPSC technology circumvents the immune-rejection problem^3^. Previous studies indicated that iPSCs resemble ESCs in terms of pluripotency, morphology, growth requirements and proliferation, markers and gene expressions, and epigenetic and functional features^2^,^4^,^5^.

Traditionally, iPSCs were generated using four factors, *Oct4 (O*), *Sox2 (S*), *Klf4 (K*), and *c-Myc (M*) (OSKM), which are now called Yamanaka factors^2^. All Yamanaka factors are transcription factors, and the detailed molecular mechanisms underlying their effects are still not fully understood. Many attempts have been made to derive iPSCs from different species and different somatic cell types by delivering the four Yamanaka factors^6^.

A growing body of research is devoted to identifying more convenient and efficient reprogramming strategies. Certain reports have indicated that iPSCs can be generated by less than four Yamanaka factors, but the cells being reprogrammed need to express some of the Yamanaka factors endogenously. For example, human umbilical vein endothelial cells (HUVECs) and hematopoietic stem cells (HSCs) already express high levels of Klf4^7^,^8^,^9^, and thus application of Oct4 and Sox2 is sufficient to reprogram HUVECs and HSCs into iPSCs^7^,^8^,^9^. Similarly, human neural stem cells (NSCs) or dermal papilla cells with high endogenous expression levels of Sox2 and c-Myc can be reprogrammed into iPSCs with Oct4 and Klf4^10^,^11^,^12^. Since NSCs also express Sox2 protein, Oct4 is sufficient to reprogram NSCs into iPSCs with low efficiency^13^,^14^. All of these studies with one or two Yamanaka factors require cells that express high endogenous levels of one or two reprogramming factors (Hester *et al*.^10^; Kim *et al*.^11^; Ho *et al*.^7^; Tsai *et al*.^12^). In fibroblast, a study has successfully reprogrammed mouse and human fibroblasts with Oct4 and Sox2^15^, and the reprogramming efficiency of iPSCs has greatly improved by certain defined small-molecule scompounds^16^. In addition, mouse fibroblast can form iPSCs with Oct4 and small-molecule compounds^17^. However, few soluble protein factors and membrane proteins have been reported to promote iPSC formation. Therefore, there is increasing interest in identifying novel reprogramming factors that are not transcription factors or chromatin modifiers.

Epithelial cell adhesion molecule (EpCAM) is a 34~40-kDa type I transmembrane protein, which is a well-known tumor-associated antigen expressed abundantly in various types of carcinoma^18^. EpCAM consists of three distinct domains; an extracellular region (EpEX), a transmembrane domain, and an intracellular domain (EpICD)^19^. We previously demonstrated that EpCAM is not only enriched in ESCs, where it regulates the four Yamanaka factors^20^, but it also plays an important role in regulating cancer-initiating abilities, self-renewal, and invasiveness in colon cancer^21^. Moreover, it has been reported that overexpression of EpCAM or EpICD decreased the expression of p53 and p21, and activated the promoter activity of Oct4 in reprogramming MEFs^4^. However, it is unknown whether EpEX can increase the efficiency of iPSC formation and whether EpCAM/EpEX can replace one or more Yamanaka factors. The functional roles of EpEX in stem cells and reprogramming processes, as well as the downstream signal(s) and receptor(s) of EpEX, remain elusive. Here, we demonstrate that EpEX and EpCAM are sufficient to reprogram fibroblasts into iPSCs with only Oct4 or Klf4. Moreover, we describe a new way of triggering reprogramming with a soluble factor and transmembrane protein, and elucidate the underlying mechanism through which EpEX increases the expressions of pluripotency genes by regulating the STAT3-HIF2α signals.

## Results

### EpCAM/EpEX enhance reprogramming efficiency in OSKM-mediated iPSCs

**Figure 1:**
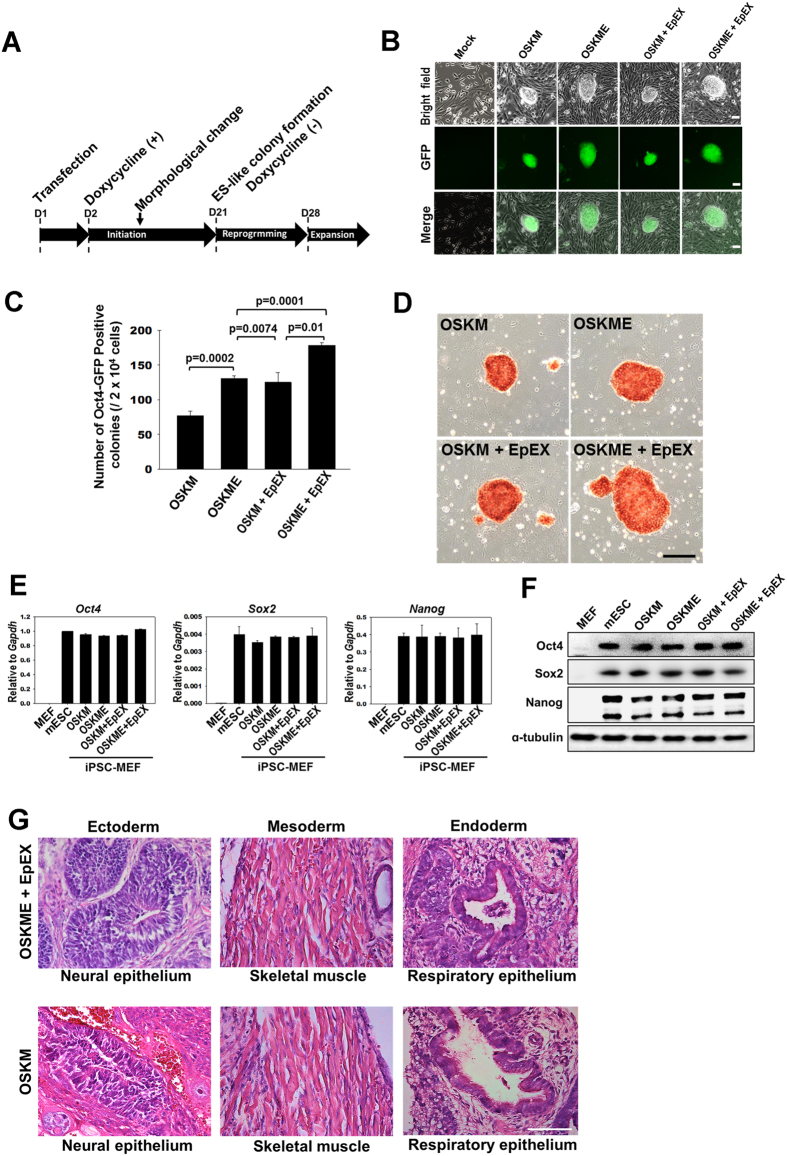
EpEX and/or EpCAM promote the reprogramming of Oct4-GFP mouse embryonic fibroblasts (MEFs) into iPSCs. (**A**) The time line of iPSC induction. The expression of exogenous reprogramming genes was induced by doxycycline from day 2–21. After induction, GFP-positive colonies were formed with either OSKM (O: Oct4; S: Sox2; K: Klf4; M: c-Myc) or OSKME (OSKM + EpCAM; E: EpCAM), with or without EpEX (soluble extracellular domain of EpCAM) treatment. (**B**) Fluorescence microscopy was used to reveal Oct4 expression in Oct-GFP iPSC colonies on day 25. Scale bar: 50 μm. (**C**) The formations of Oct4-GFP iPSCs was evaluated by examining colony numbers on day 25 under fluorescence microscopy. (**D**) Alkaline phosphatase staining was performed to examine undifferentiated iPSCs. Scale bar: 50 μm. (**E**) Expression of pluripotency genes in iPSCs. Q-PCR analysis was used to show that iPSCs express the endogenous pluripotency genes *Oct4, Sox2*, and *Nanog*. Parental MEFs were used as a control (n = 3). (**F**) Western blot analysis was performed to reveal that the iPSCs express Oct4, Sox2, and Nanog protein (n = 3). (**G**) The pluripotency of iPSCs was examined by teratoma formation. Mice were injected with OSKM and OSKME + EpEX iPSCs (n = 3). Scale bar: 50 μm.

We examined if EpEX and EpCAM can increase reprogramming efficiency during OSKM-mediated iPSC formation. We used the *PiggyBac* transposon system to avoid virus-mediated insertional mutagenesis^22^,^23^. Nuclear electroporation was used to transfect *Oct4 (O*), *Sox2 (S*), *Klf4 (K*), *c-Myc (M*), and *EpCAM (E*) genes, which were induced by doxycycline in mouse embryonic fibroblasts (MEFs) with Oct4 promoter-driven GFP. The transfectants are henceforth referred to as ‘OSKM’, ‘OSKME’, ‘OSKM + EpEX’, and ‘OSKME + EpEX’. Figure 1A illustrates the iPSC generation procedures. GFP-positive colonies (indicating endogenous Oct4 expression) were observed after reprogramming (Fig. 1B). Compared to OSKM, the reprogramming efficiency was elevated by over 1.6 fold in OSKME and OSKM + EpEX, and 2.4 fold in OSKME + EpEX (Fig. 1C and Table 1).

The undifferentiated status of all iPSCs was confirmed by alkaline phosphatase (AP) staining (Fig. 1D). RNA (*Oct4, Sox2, Nanog*) and protein (Oct4, Sox2, Nanog) amounts in iPSCs were comparable to those in mouse ESCs (Fig. 1E,F). Next, iPSC pluripotency was examined by teratoma formation. The iPSCs (OSKM and OSKME + EpEX groups) differentiated into three germ layers: neural epithelium (ectoderm), skeletal muscle (mesoderm), and respiratory epithelium (endoderm) (Fig. 1G).

### EpCAM/EpEX with either Oct4 or Klf4 can induce iPSCs

**Figure 2:**
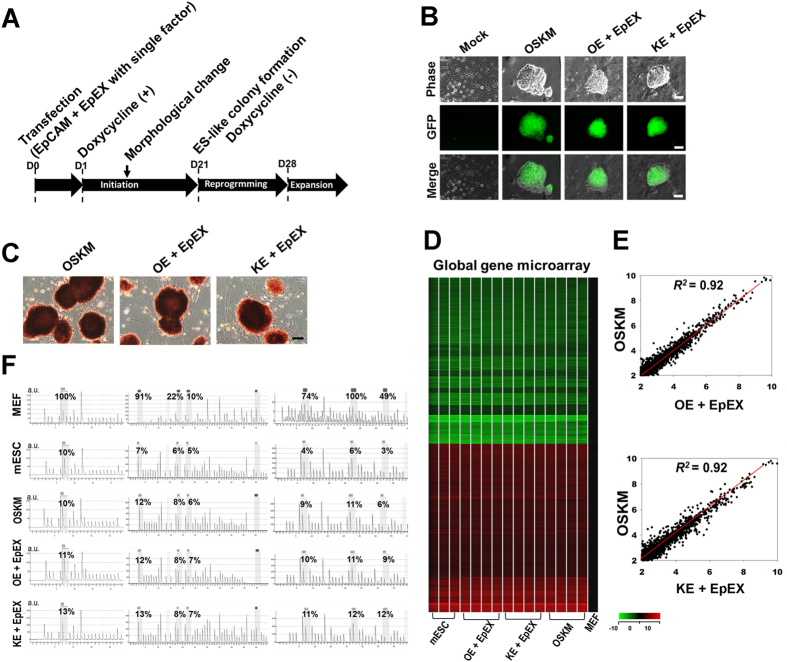
EpEX and EpCAM in combination with only Klf4 or Oct4 are sufficient to reprogram MEFs into iPSCs. (**A**) Protocol for iPSC generation. (**B**) Fluorescence microscopy was used to visualize iPSC colonies transfected with Oct4 or Klf4 and EpCAM, with or without EpEX treatment (1 μg/mL). The OSKM group served as a positive control. Scale bar: 50 μm. (**C**) Alkaline phosphatase staining was performed to examine the stemness status of iPSCs. Scale bar: 50 μm. (**D**) RNA microarray analysis of the global gene profile of iPSCs of the OSKM, OE + EpEX, and KE + EpEX groups. Three different clones from mouse ES cells (mESCs) (n = 3), four different clones from each iPSC group (n = 4). Parental MEFs were used as a control. The heat map indicates the global gene expression pattern for each group. (**E**) Scatter plot comparing global gene expression profiles of iPSCs generated by OSKM and OE + EpEX or KE + EpEX. (**F**) DNA methylation status of the Oct4 promoter. Disulfide conversion and pyrosequencing were performed to detect DNA methylation of the Oct4 promoter in the indicated groups. The percentage represents the methylation level at the indicated position.

Next, we examined if EpEX and/or EpCAM can substitute for multiple Yamanaka factors in iPSC formation. Figure 2A illustrates iPSC protocol. We transfected cells with doxycycline-inducible EpCAM, with or without a single Yamanaka factor (Oct4, Sox2, or Klf4), to generate four groups: OE, SE, KE, and E. We omitted c-Myc due to its oncogenicity and dispensability for reprogramming^2^,^24^,^25^. GFP colony formation was not observed under any condition (Table 2).

We treated these cells with EpEX (designated as EpEX, O + EpEX, S + EpEX, K + EpEX, OE + EpEX, SE + EpEX, KE + EpEX, and E + EpEX). Colonies were found in the KE + EpEX, OE + EpEX, and OSKM groups (Fig. 2B and Table 2). iPSC formation efficiency was 0.05% for the OE + EpEX group, 0.03% for the KE + EpEX group, and 0.18% for the OSKM group (Table 2). Positive AP staining was observed for each group (Fig. 2C).

### Characterization of iPSCs by *in vitro* and *in vivo* assays

**Figure 3:**
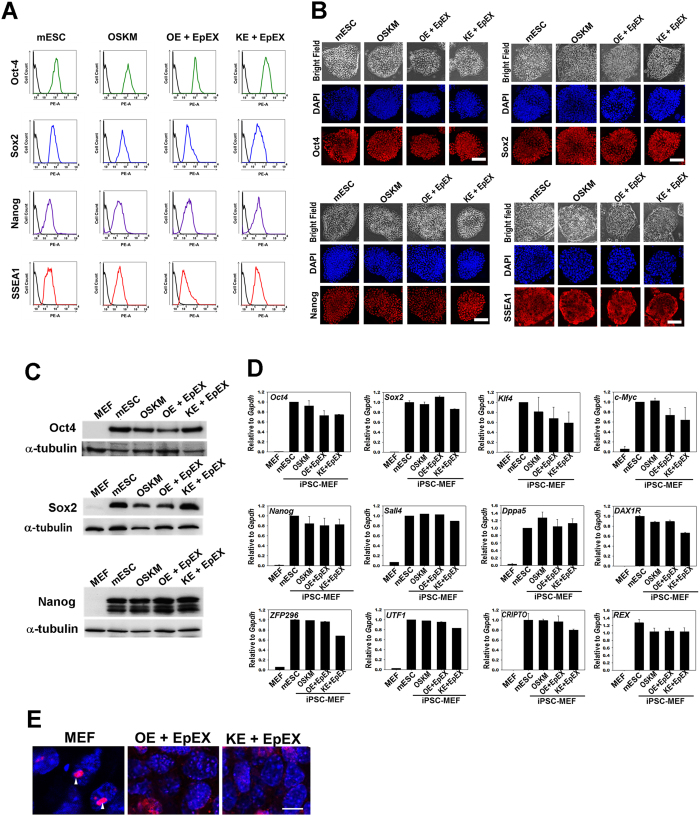
Characterization of iPSCs *in vitro*. (**A**) Flow cytometry was performed to detect Oct4, Sox2, Nanog, and SSEA1 (n = 3). (**B**) Immunofluorescent staining was performed for the detection of Oct4, Sox2, Nanog, and SSEA1 by confocal microscopy (n = 3). Scale bar: 50 μm. (**C**) Western blotting for the detection of Oct4, Sox2, and Nanog (n = 3). (**D**) Q-PCR analysis (n = 3). Q-PCR analysis was used to show that the iPSCs express endogenous pluripotency genes, including *Oct4, Sox2, Klf4, c-Myc, Nanog*, Spalt-Like Transcription Factor 4 (*Sall4*), dosage-sensitive sex reversal, adrenal hypoplasia critical region, on chromosome X, gene 1 (*DAXR1*), developmental pluripotency-associated 5 (*Dppa5*), zinc finger protein (*ZFP*), undifferentiated embryonic cell transcription factor (*UTF*), cysteine-rich PDZ-binding protein (*CRIPTO*), reduced expression (*REX*). The parental MEF group was used as a control. (**E**) me3H3K27 immunostaining of MEFs and iPSCs. Red color indicates the staining of me3H3k27. Blue color indicates the DAPI staining. Scale bar: 10 μm.

Global gene expression patterns of these mouse iPSCs and ES cells were similar (Fig. 2D). Scatter plots indicated that global gene expression profiles of KE + EpEX and OE + EpEX iPSCs were similar to that of OSKM iPSCs (Fig. 2E). The DNA methylation state of *Oct4* promoter in iPSCs was similar to that in ESCs, while *Oct4* promoter was highly methylated in MEFs (Fig. 2F). Expression of Oct4, Sox2, Nanog, and SSEA1 (surface marker of mouse ES cells) in KE + EpEX, OE + EpEX iPSCs and mouse ES cells were comparable (Fig. 3A). Immunofluorescent staining revealed strong positive signals for Oct4, Sox2, Nanog, and SSEA1 in each iPSC group, comparable to those of mouse ESCs (Fig. 3B). Protein levels of Oct4, Sox2, and Nanog were similar in all iPSCs and mouse ESCs (Fig. 3C). OE + EpEX and KE + EpEX iPSCs expressed many pluripotent genes (see Figure legend of Fig. 3D) at levels equivalent to those in the mouse ES cell line (Fig. 3D). In addition, the loss of me3H3K27 (red) indicates the X chromosomes reactivation in iPSCs (Fig. 3E).

**Figure 4:**
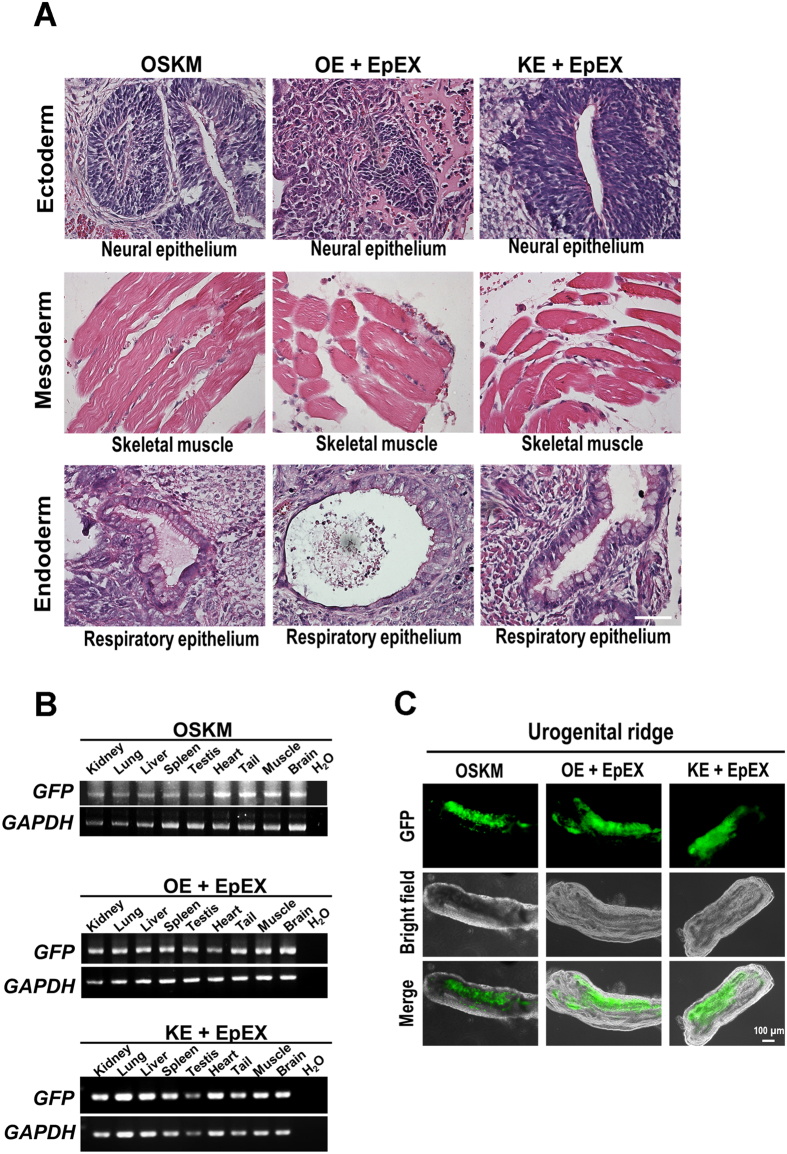
Characterization of iPSCs *in vivo.* (**A**) Teratoma formation (n = 3). Three germ layers (ectoderm, endoderm, and mesoderm) were detected in all iPSCs. Scale bar: 50 μm. (**B**) Genotyping of chimeras. Single iPSC clones were picked and amplified for each group. We injected 6–11 iPSCs into a 3.5 day-old blastocyst (n = 3). The GFP signals represent chimerism in multiple tissues. (**C**) The urogenital ridge in 13.5 day embryos was observed under fluorescence microscopy. Oct4 signals from iPSCs were detected in the urogenital ridge (n = 3).

We examined the pluripotency of KE + EpEX and OE + EpEX iPSCs based on teratoma formation (Fig. 4A). We injected KE + EpEX and OE + EpEX iPSCs into 3.5 day-old blastocysts, and detected the GFP cassette in various organs of chimeras by genotyping (Fig. 4B). Germ-line transmission of the cassette was also confirmed by GFP positive signals within the gonadal ridge (13.5 day-old embryos) (Fig. 4C).

### EpEX/EpCAM-induced STAT3 signal during reprogramming

**Figure 5:**
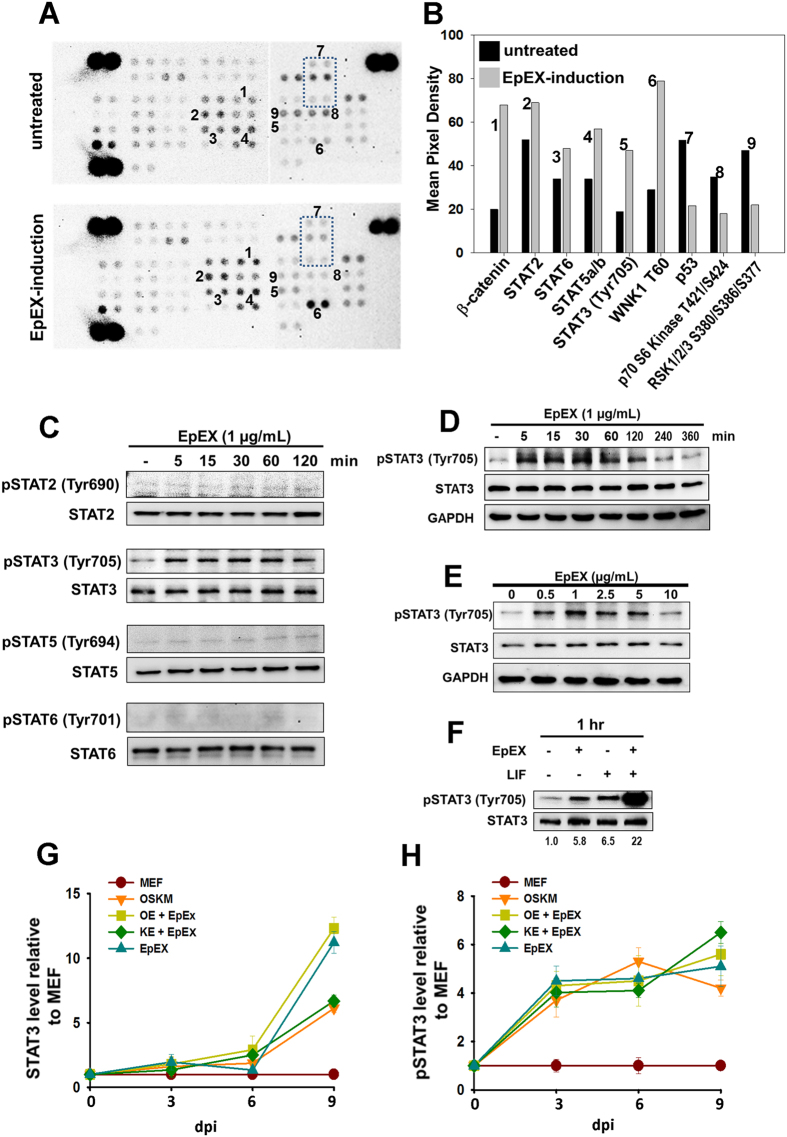
EpEX/EpCAM-mediated iPSC reprogramming through STAT3 signal. (**A**) The phospho-kinase array detects phosphorylated proteins in untreated and EpEX-treated MEFs. (**B**) Quantification by mean pixel density revealed that six phosphorylated proteins are regulated by EpEX. (**C**) MEFs are stimulated by EpEX in a time-dependent manner. MEFs were treated by EpEX (1 μg/mL) for 5, 15, 30, 60, or 120 min. After treatment, cells were harvested, and phosphorylation of STAT proteins was detected by Western blotting (n = 3). (**D**,**E**) MEFs were stimulated by EpEX in dose- and time-dependent manners. MEFs were treated with EpEX at concentrations of 0, 0.5, 1, 2.5, 5, 10 μg/mL for 15 min, or with EpEX (1 μg/mL) for 5, 15, 30, 60, 120, 240, 360 min. After treatment, cells were harvested, and the phosphorylation of STAT3 was detected by Western blotting (n = 3). (**F**) MEFs were stimulated with EpEX (1 μg/mL) and/or LIF (1 μM) for 1 h. The phosphorylation of STAT3 (Tyr 705) was examined by Western blotting (n = 3). (G and H) MEFs were transfected with OSKM, OE, and KE, and treated with EpEX. After transfection, cells in each group (including control MEFs) were stimulated with doxycycline (1.5 μg/mL) in iPSC medium (DMEM/F12 with stem cell knockout serum, without LIF) and harvested on days 3, 6, or 9. STAT3 and phopho-STAT3 protein expression was detected by Western blotting (n = 3). Kinetic expression was shown after quantification.

Phospho-kinase array revealed that EpEX significantly increased phosphorylated STAT proteins, phosphorylated β-catenin, and WNK lysine-deficient protein kinase 1 (WNK1) (Fig. 5A,B). Moreover, EpEX decreased the phosphorylation of p53, Ribosomal protein S6 kinase beta-1 (p70 S6 kinase), and ribosomal s6 kinase 1/2/3 (RSK1/2/3) (Fig. 5A,B). Western blotting revealed that EpEX significantly activates STAT3 (tyrosine 705) phosphorylation, with modest effects on phospho-STAT2, STAT5, and STAT6 (Fig. 5C).

The dose- and time-dependent effects of EpEX on STAT3 activation were examined by Western blotting. STAT3 phosphorylation (tyrosine 705) was significantly induced by EpEX within 5 minutes and gradually decreased after 4 hours (Fig. 5D). The optimal dose of EpEX for STAT3 activation is 0.5–5 μg/ml (Fig. 5E). Since LIF is known to maintain pluripotent embryonic stem cell (ESC) self-renewal via STAT3^26^, we also tested the effects of EpEX and/or LIF on STAT3 phosphorylation in MEFs. In the absence of LIF, EpEX can activate STAT3 phosphorylation to a level comparable to that of LIF stimulation (Fig. 5F). Co-incubation of EpEX and LIF significantly boosted STAT3 phosphorylation, indicating that EpEX can induce STAT3 phosphorylation in the absence of LIF, and these signals may differ from the LIF/LIF receptor signaling; therefore, EpEX and LIF have a significant synergistic effect on STAT3 activation (Fig. 5F). Moreover, we found the level of total STAT3 and phospho-STAT3 were increased in OE + EpEX, KE + EpEX, and OSKM reprogramming on days 3, 6, and 9, as compared to non-treated MEFs (Fig. 5G,H). Each group was maintained in iPSC medium without LIF. Thus, we demonstrated that EpEX or EpEX/EpCAM may promote reprogramming through STAT3 signaling. Following up on the finding that EpEX-induced STAT3 signaling and EpCAM/EpEX can help initiate reprogramming, we proceeded to examine whether EpCAM is critical for iPSC maintenance by knocking down EpCAM expression with two EpCAM shRNA clones in iPSCs. EpCAM depletion decreases Oct4, Nanog, Klf4, and phosphorylated STAT3, but not total STAT3 (Supplementary Fig. S2A). Thus, EpCAM is critical for maintaining the levels of pluripotent factors as well as STAT3 signaling. Moreover, WP1066, a STAT3 inhibitor, significantly suppressed expression of Oct4, Klf4, and Nanog, indicating that STAT3 is critical for pluripotency maintenance in iPSCs (Supplementary Fig. S2B). Taken together, EpEX promotes STAT3 activation in MEFs, and EpCAM promotes pluripotent gene expression via STAT3 activation.

### EpEX activates HIF2α via STAT3 during iPSC formation

**Figure 6:**
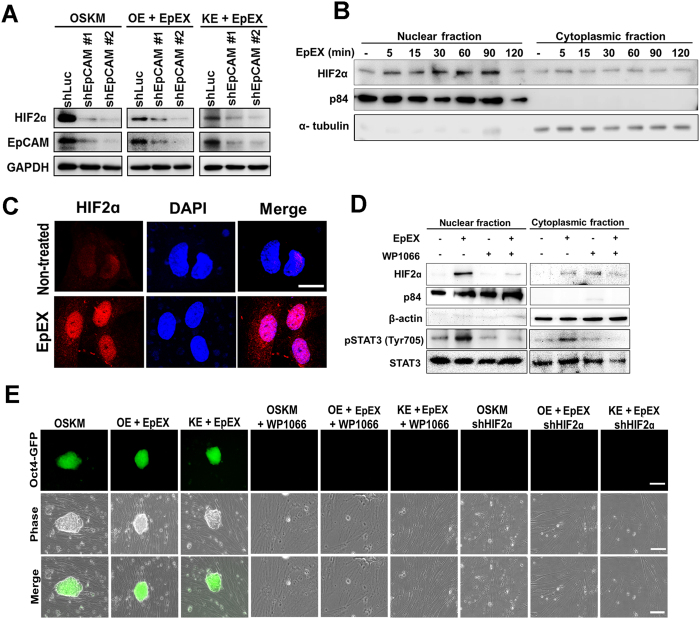
EpEX and EpCAM activate a STAT3-HIF2α signal for EpEX/EpCAM-mediated iPSC formation. (**A**) iPSCs were infected with two different EpCAM shRNAs (two clones, #1 and #2). The protein expressions of EpCAM and HIF2α were detected by Western blotting. (**B**) MEFs were stimulated by EpEX (1 μg/mL) at the indicated times. Nuclear-translocation was detected with a specific antibody against HIF2α (n = 3). (**C**) Immunofluorescence staining was performed to detect subcellular localization of HIF2α. Nuclei were stained with DAPI. Scale bar: 10 μm. (**D**) MEFs were treated with STAT3 inhibitor (WP1066, 10 μM), and then stimulated with EpEX for 30 min. The nuclear-translocation of HIF2α was detected by Western blotting with anti-HIF2α antibody (n = 3). (**E**) iPSC morphology was observed at day 20 after induction. Reprogramming of Oct4-GFP MEFs was induced by transfection of OSKM, OE + EpEX, and KE + EpEX with or without STAT3 inhibitor WP1066, or HIF2α shRNA (n = 3). Scale bar: 50 μm.

HIF2α (hypoxia inducible factor 2α) supports Oct4 in mouse ESCs^27^ and enhances stemness by binding to Nanog promoter in human ESCs^28^. IL6-STAT3-HIF2α signaling contributes to angiogenesis in ovarian cancer^29^. HIF2α has been found to enhance the levels of Oct4, Sox2, and Nanog to maintain pluripotency^30^. In addition, EpCAM expression is required for HIF2α expression (Fig. 6A). Thus, we hypothesized that HIF2α may participate in EpEX-STAT3-mediated reprogramming. We have shown that EpEX induced nuclear translocation of HIF2α in fibroblasts (Fig. 6B,C) and that WP1066 (STAT3 inhibitor) abolished the EpEX-induced HIF2α nuclear translocation (Fig. 6D). We have confirmed that the underlying mechanism of WP1066 is through the detection of the inhibited STAT3 phosphorylation (Fig. 6D). These results suggested that EpEX-induced HIF2α nuclear translocation is regulated by STAT3. Furthermore, iPSCs did not form when OSKM, OE + EpEX, or KE + EpEX groups were treated with WP1066 or HIF2α shRNA (two clones, #1 and #2) (Fig. 6E).

Thus, STAT3 and HIF2α are crucial for both pluripotency regulation and maintenance in iPSCs. In addition, we detected the expression of pluripotent factors (Oct4, Sox2, Klf4, Nanog, EpCAM) during reprogramming (Supplementary Fig. S1), the expression of EpEX in conditioned medium, and the expression of EpCAM in cells at day 3, 6 and 9 (Supplementary Fig. S3). The results demonstrate the EpEX and EpCAM promote iPSC reprogramming through activation of STAT3 and translocation of HIF2α.

## Discussion

The study reveals that (i) EpCAM and EpEX increase the efficacy of iPSC generation, (ii) EpEX/EpCAM can form iPSCs with just one Yamanaka factor (Klf4 or Oct4), (iii) EpEX/EpCAM promotes iPSC formation through STAT3-HIF2α signaling. This is the first report to show that Yamanaka factors can be replaced by transmembrane protein, EpCAM, and its extracellular domain (EpEX).

EpCAM is essential for the expression of Oct4, Sox2, Klf4, c-Myc and SSEA-1, and alkaline phosphatase activity^20^,^31^. Here, we found that EpCAM and EpEX enhance iPSC formation efficiency, while inhibition of EpCAM down-regulates the expression of Oct4, Sox2, Klf4, c-Myc (Supplementary Fig. S2A). We are first to show that fibroblasts can be reprogrammed by a single Yamanaka factor (either Oct4 or Klf4) in combination with EpEX and EpCAM. This suggests that EpEX and EpCAM can replace the function of Sox2 and Oct4/Klf4 in reprogramming.

Previously, it has been shown that elevation of EpCAM expression enhances tumor sphere formation and tumor initiation^21^. In 2009, Maetzel *et al*. proposed the novel concept that activation of the EpCAM surface-to-nucleus signaling transduction pathway in cancer occurs through regulated intracellular proteolysis^32^. Upon proteolytic cleavage, the extracellular domain of EpCAM is cleaved by activated ADAM17 (TACE) to release soluble EpEX. Subsequently, EpICD is cut by γ-secretase. The increased release of EpEX enhances the production of EpICD and regulates the expression of reprogramming factors^21^. EpICD associates with β-catenin and cofactor FHL2 (four and a half LIM domain protein 2) to form a transcriptome complex that translocate into the nucleus to modulate the transcriptional activity of target genes, such as c-Myc and promote tumor progression^32^,^33^.

EpEX was recently identified as the secreted extracellular part of the transmembrane protein, EpCAM^32^. We previously showed that EpCAM is enriched in human ESCs and that EpICD binds to Yamanaka factor promoters^20^. Moreover, EpCAM overexpression promotes iPSC formation via inhibition of p53 and p21^4^. The function and signals of EpEX has never been reported in reprogramming or ESC renewal. We found that iPSCs can be formed by using EpCAM and EpEX, combined with one Yamanaka factor, Oct4 or Klf4 (Figs 2,3 and 4) and that EpEX increases the efficiency of iPSC formation (Fig. 1). This prompts us to suggest that EpEX and EpCAM may activate signaling to initiate reprogramming. Since iPSCs were not formed in the SE + EpEX group but did form in the OE + EpEX and KE + EpEX groups, we propose that EpEX/EpCAM cannot replace the functions of both Oct4 and Klf4, but can substitute for Sox2, or help increase Sox2 levels during reprogramming. Previously, it was reported that the STAT3 signal up-regulates Sox2 expression^34^, thus we hypothesize that EpEX/EpCAM may up-regulate Sox2 by activating STAT3.

It has been shown that a small molecule inhibitor with several defined factors can replace Oct4 and Sox2^35^,^36^,^37^. E-cadherin induces reprogramming with three Yamanaka factors (Sox2, Klf4, and c-Myc), without Oct4^38^. However, unlike EpCAM/EpEX, none of these signals can concurrently replace both Oct4 and Sox2, and reprogram fibroblasts with Klf4 alone. EpICD binds to Yamanaka factor promoters in human ESCs^20^, while EpEX triggers the production of EpICD as well as regulates the expression of reprogramming factors in colon cancer cells^20^,^21^,^32^; therefore, endogenous reprogramming factors may be up-regulated in OE + EpEX and KE + EpEX iPSCs. Soluble EpEX enhances the cleavage of EpEX and induces EpICD signaling in an autocrine or paracrine manner, and promotes tumor initiation and progression^21^. Moreover, we showed that the activity of TACE in iPSCs and mouse ESCs is highly induced compared to that of MEF (Supplementary Fig. S4A). Interestingly, we detected the activity of TACE in OSKM, OE + EpEX, KE + EpEX, OE + TACE, and KE + TACE groups, and found the effects of adding TACE to be similar to that of EpEX treatment during early induction of reprogramming on day 3 (Supplementary Fig. S4B). EpEX induces the phosphorylation and activation of TACE in MEFs (Supplementary Fig. S4C,D). In addition, we investigated whether treatment of MEFs with TACE can result in the shedding of EpEX and may have a similar effect for reprogramming. We transfected EpCAM with Oct4 or Klf4, and treated with TACE (5 ng/mL). Results showed that iPSCs did not form in OE + TACE, and KE + TACE groups on day 20 (Supplementary Fig. 7). Such result indicated that the treatment of TACE may lead to EpEX shedding. Such level of EpEX, however, is likely not sufficient for the triggering of reprogramming signals.

Furthermore, we found that EpEX induces STAT3 phosphorylation in MEFs, suggesting that EpEX may serve as a cytokine. Thus, we suppose that EpEX can activate receptors and may initiate reprogramming through the downstream signal transduction. We showed that EpEX can induce the phosphorylation of EGFR, PDGFR, HER2, and Axl by phospho-kinase array (Supplementary Fig. S5A,B). Recent study demonstrated that EGFR and PDGFR play important roles in reprogramming^39^,^40^, which is consistent with our previous results and may explain the efficacy of treatment of EpEX. In addition, we considered the concentration of EpEX, which we optimized in iPSC usage, to be much higher than that of the shedding of EpEX. Therefore, treatment of EpEX may enhance these signaling and drive the initiation of iPSC generation. Moreover, we supposed that since MEF does not expresses EpCAM endogenously, the efficacy of TACE treatment may not have influence on triggering the initiation processes in the beginning of reprogramming, while EpEX treatment may exert potent impact on the initiation of reprogramming through triggering and enhancing these signaling.

Alternatively, we confirmed the importance of EpICD during early induction of reprogramming and delivered EpICD-truncated EpCAM and Oct4 or Klf4 and alone with the treatment of EpEX in MEFs. The construction of EpICD-truncated EpCAM is confirmed by Western blotting (Supplementary Fig. S6). We did not observe the iPSC formation in the group of EpICD-truncated EpCAM with Oct4 or klf4 with EpEX treatment on Day 20 (Supplementary Fig. S7), which indicated the necessity of EpICD.

A few types of factors were identified to be able to substitute for Oct4 during reprogramming, such as GATA3, GATA6 and SOX7 (lineage specifiers of mesendoderm)^35^ or substitute for Sox2, ZNF521 (ectoderm specifier)^36^. Additionally, maternal transcription factor Glis1 and Nr5a2 can replace Oct4 for reprogramming^41^,^42^. E-cadherin has also been reported to induces reprogramming with three of the Yamanaka factors (Sox2, Klf4, and c-Myc), without the need for Oct4^38^. Interestingly, E-cadherin and other cell-adhesion molecules also have functional roles in the mesenchymal to epithelial transition (MET)^43^,^44^, which is crucial for iPSC reprogramming. However, with the exception of EpCAM and EpEX, none of these can concurrently replace Oct4 and Sox2 signals and reprogram fibroblast with only Klf4. We also proposed that EpCAM/EpEX is not sufficient to replace both Oct4 and Klf4 and that it may act as an ectoderm driver due to the failure of iPSC formation in SE + EpEX group. Another hypothesis is that EpEX and EpCAM promote iPSC formation through other routes. For example, EpCAM is expressed in epithelial cells^18^. Thus, it will be interesting to determine if EpCAM and EpEX promote MET to facilitate iPSC formation. Here, we describe a novel pathway: EpEX/EpCAM/STAT3/HIF2α signaling. STAT3 signaling is critical for mouse ESCs and naïve pluripotent stem cells, and is activated by LIF^26^; EpEX induces STAT3 phosphorylation in MEF in the absence of LIF (Fig. 5C–E). The addition of both EpEX and LIF synergistically increase phospho-STAT3, as compared to cells treated with only EpEX or LIF (Fig. 5F). Total and phosphorylated STAT3 were up-regulated during early induction of reprogramming by EpEX in the absence of LIF (Fig. 5G,H).

HIF2α also mediates pluripotency by forming a complex with Oct4 and by enhancing its activity^45^. Therefore, EpEX may induce the nuclear-translocation of HIF2α and promote the expressions of target genes. Interestingly, nuclear HIF2α expression requires EpEX and STAT3 (Fig. 6A–D). HIF2α interacts with a glycolytic enzyme, pyruvate kinase isozyme M2 (PKM2), to promote Oct4 transcription^28^. PKM2 overexpression enhances Oct4 levels and supports self-renewal in the absence of LIF in ESCs^46^. Thus, EpEX may also activate PKM2/HIF2α, whereby up-regulating Oct4. Additionally, EpICD is critical for pluripotency and self-renewal in human ESCs^20^. We will determine whether EpEX promotes EpICD signaling and whether EpICD associates with HIF2α and STAT3 to form a transcription interactome to promote reprogramming.

In conclusion, our results demonstrate that EpCAM/EpEX activates the STAT3-HIF2α complex in iPSC reprogramming. EpEX is particularly advantageous in iPSC generation since it reduces the risk of insertional mutagenesis. Such findings provide a new strategy for iPSC formation and may contribute to future regenerative medicine techniques.

## Methods

### Feeder cell isolation

For MEF isolation, we isolated uteri from mice on day 13.5 of pregnancy. The head, feet, tail, and organs of the fetuses were removed. The remaining bodies were washed in fresh PBS (contain 1% v/v penicillin-streptomycin), minced with scissors, and incubated with 0.5% Trypsin-EDTA at 37 °C for 20 min. Cells were collected by centrifugation (200 g for 5 min at 4 °C) and resuspended in fresh media. Cells were cultured on 100 mm dishes at 37 °C with 5% CO_2_.

### Plasmid construction

PB-TET-OSKM was purchased from Addgene (20959; Addgene, Cambridge, MA, USA). The OSKM segment of PB-TET-OSKM was removed using PCR-based gene deletion^41^, and Oct4, Sox2, Klf4, or EpCAM were then inserted respectively into the PB-TET backbone by restriction enzyme cloning. Each construct was confirmed by DNA sequencing. The N-terminal 289 amino acid extracellular region and transmembrane domain of mouse EpCAM was amplified by Polymerase chain reaction (PCR) and subcloned into pcDNA3.1/V5-His A and PB-TET expression vector. The HIF-2α respective DNA fragments were amplified by using HIF-2α cDNA (GenScript) as a template. The HIF-2α PCR fragments were flanked by a KpnI and XhoI restriction site, cleaved with KpnI/XhoI, and then cloned into the corresponding sites of the pcDNA3.1/V5-His A expression vector (Invitrogen).

### iPSC generation

The iPSC generation was performed as described^47^. In brief, MEFs with Oct4 promoter driven GFP were isolated from E13.5 mice (B6.Cg-Gt (ROSA) 26Sortm1 (rtTA*M2) Jae/J; B6.129S4-POU5f1tm2Jae/J) (Jackson Lab) were used to generate iPSCs. We used MEFs within four passages and cultured with high-glucose DMEM, 10% FBS (Invitrogen), 1 mM L-glutamine (Invitrogen), 10 mM nonessential amino acids (Invitrogen), 10 mM penicillin/streptomycin (Invitrogen), and 10 mM HEPES (Invitrogen). Oct4-GFP MEFs (1 × 10^6^) were nucleofected with 2.5 μg of *PiggyBac* helper plasmids and 2.5 μg of PB-TET-OSKM (20959; Addgene)^41^, or with different combinations of PB-TET-Oct4, PB-TET-Sox2, PB-TET-Klf4, or PB-TET-EpCAM (listed in Tables 1 and 2). After 24 h, 1.5 μg/ml doxycycline was added to induce the expression of reprogramming factors for the entire course of reprogramming. The culture medium was first changed 48 h after nucleofection; followed by daily change until the formation of visible colonies strongly expressing GFP (usually after 21 d). To estimate the reprogramming efficiency, we counted the number of GFP positive colonies (Oct4 positive) and divided them by the numbers of seeding cells (20000 cells/well). Wild type MEFs were isolated from C57BL/6 J mice at embryonic day 13.5 (E13.5) and were treated with mitomycin C (10 μg/mL) (MDBio, Inc. Taipei, Taiwan). The mouse iPSC and ESC medium consisted of Dulbecco’s modified Eagle’s medium/F-12 (DMEM/F12, Invitrogen, Carlsbad, CA, USA) with 20% knockOut serum replacement (Invitrogen), 1 μM LIF (Millipore, Billerica, MA, USA), 1 mM β-mercaptoethanol (Sigma-Aldrich, St. Louis, MO, USA), 10 mM nonessential amino acids (Invitrogen), and 1 mM L-glutamine (Invitrogen). After 24 h, doxycycline (1.5 μg/mL) (Sigma-Aldrich) was added to induce the expression of reprogramming factors for 21 days. The culture medium was changed daily after nucleo-transfection.

### Alkaline phosphatase assay

Alkaline phosphatase staining was performed according to the manufacturer’s recommendations (System Biosciences, SBI; AP100 R-1). Briefly, cells were fixed in 4% paraformaldehyde (PFA) for 5 min, and then washed with PBS. Cells were stained with staining solution for at least 1 min, and the results were observed under a reflected-light microscope (Axio Scope Vario HAL 100, Carl Zeiss, Inc, Jena, Germany). Images were captured using a ZEISS cool SNAP camera (Carl Zeiss, Inc).

### Quantitative real time RT-PCR

Total RNA was extracted using TRI reagent (Invitrogen, CA, USA), and 5 μg of total RNA were reverse transcribed into cDNA using oligo (dT) primer (Fermentas, Glen Burnie, MD, USA) with SuperScript III reverse transcriptase (Invitrogen). With the cDNA, quantitative real time RT-PCR (QRT-PCR) was performed using the Light Cycler® 480 SYBR Green I Master kit (Roche Applied Science, Indianapolis, IN) and the LightCycler480 System (Roche Applied Science). The gene expression levels of each sample were normalized to the expression levels of glyceraldehyde 3-phosphate dehydrogenase (GAPDH).

Primers are listed as follows: Oct4, forward: 5′-TCTTTCCACCAGGCCCCCGGCTC-3′; reverse: 5′-TGCGGG CGGACATGGGGAGATCC-3′; Sox2, forward: 5′-GGTTACCTCTTCCTCCCA CTCCAG-3′; reverse: 5′-TCACATGTGCGACAGGGGCAG-3′; KLF4, forward: 5′- GCGAACTCACACAGGCGAGAAACC-3′; reverse: 5′-TCGCTTCCTCTTCCT CCGACACA-3′; c-Myc, forward: 5′-TGACCTAACTCGAGGAGGAGCTGGAATC -3′; reverse: 5′-AAGTTTGAGGCAGTTAAAATTATGGCTGAAGC-3′; Nanog, forward: 5′-AAAAAGCAGGCTCTGACATGAGTGTGGGTCTT-3′; reverse: 5′- AGAAAGCTGGGTAAGTCTCATATTTCACCTGG-3′; Sall4, forward: 5′-CAC CATGTCGAGGCGCAAGCAGGCGAAGCC-3′; reverse: 5′-TTAGCTGACAGC AATCTTATTTTCCTCCAG-3′; Dnmt3, forward: 5′-AAAAAGCAGGCTCAA TGGGTTCCCGGGAGACA-3′; reverse: 5′-AGAAAGCTGGGTCCTGGTTCT AGGAAAGACTT-3′; Dppa5, forward: 5′-AAAAAGCAGGCTGGA TGATGGTGACCCTCG TG-3′; reverse: 5′-AGAAAGCTGGGTCTGCATCCAGGT CGGAGAC-3′; DAX1, forward: 5′-TGCTGCGGTCCAGGCCATCAAGAG-3′; reverse: 5′-GGGCACTGTTCAGTTCAGCGGATC-3′; ZFP296, forward: 5′-CCA TTAGGGGCCATCATCGCTTTC-3′; reverse: 5′-CACTGC TCACTGGAGGGGGCTTGC-3′; UTF1, forward: 5′-GGATGTCCCGGTGAC TACGTCTG-3′; reverse: 5′-GGCGGATCTGGTTATCGAAGGGT-3′; CRIPTO, forward: 5′-ATGGACGCAACTGTGAACATGATGTTCGCA-3′; reverse: 5′-CTTTGAGGTCCTGGTCCATCACGTGACCAT-3′; REX, forward: 5′-ACG AGTGGCAGTTTCTTCTTGGGA-3′; reverse: 5′-TATGACTCACTTCCA GGGGGCACT-3′.

### Western blot analysis and Phospho-Kinase Array

Western blotting was performed as previously described^48^. Cells were lysed in lysis buffer (150 mM NaCl, 50 mM Tris-HCl (pH 7.4), 1% Nonidet P-40) containing a protease inhibitor mixture (Roche Applied Science). Nuclear fraction and cytoplasmic fraction were separated by Nuclear/Cytosol Fractionation Kit according to the manufacturer’s instructions (BioVision Inc., Milpitas, CA, USA). Protein samples were separated by SDS-PAGE under denaturing conditions, and transferred to a PVDF membrane (Millipore). PVDF membranes were incubated with primary antibodies includes anti-Oct4 monoclonal antibody (ab107156, Abcam, Oxford, UK), anti-Sox2 monoclonal antibody (ab107156, Abcam), and anti-Nanog antibody (ab107156, Abcam), anti-phopho-STAT3 monoclonal antibody (ab94768, Abcam), anti-HIF2α antibody (NB100-122, Novus Biologicals, Cambridge, UK), anti-STAT3 (Catalog No. 9139, Cell Signaling Technologies), anti-EpEX (G8.8, eBioscience), anti-EpICD (E144, Abcam) anti-α-tubulin (1:5000; Sigma-Aldrich) and anti-GAPDH antibody (GeneTex, Irvine, CA, USA). Tubulin and GAPDH are the loading controls. Membranes were washed three times, and then incubated with horseradish peroxidase (HRP)-conjugated goat anti-mouse or anti-rabbit IgG (1:5000; Jackson ImmunoResearch, USA) for one hour. Finally, membranes were washed three more times, and subsequently developed using Chemiluminescence Reagent Plus (Thermo Fisher Scientific, Runcorn, UK). The Phospho-Kinase Array Kit (Proteome Profiler Antibody Array, R&D Systems) was used according to the manufacturer’s instructions.

### DNA microarray

Total RNA from ES cells, iPSCs, or MEFs were isolated using RNeasy® Plus Mini Kit (QIAGEN). RNA templates were then reverse-transcribed to cDNA and labeled with Cy3. Samples were hybridized to a mouse expression array 430 (51100, Affymetrix, Santa Clara, CA, USA) according to the manufacturer’s protocol. Arrays were scanned with a Microarray Scanner System. Data were analyzed using GeneSpring GX software (Agilent, Santa Clara, CA, USA).

### Flow cytometry analysis

Cells were dissociated with 0.25% trypsin-EDTA (1 mM) (Invitrogen) for 3 min, washed with fluorescence-activated cell sorting buffer (FACS buffer, PBS containing 1% fetal bovine serum), fixed in 4% PFA, and then permeabilized with 0.1% Triton X-100 in PBS. Subsequently, cells were stained with Oct4, Sox2, Nanog, and SSEA1 antibodies (1:100) (ab107156, Abcam, UK), washed and suspended in FACS buffer, and incubated with secondary antibody (1:200; Jackson ImmunoResearch) for 60 min at room temperature. Flow cytometry analysis was performed with a BD FACSCanto II flow cytometer (BD Biosciences, CA, USA).

### Immunofluorescence staining

Feeder MEFs were seeded first onto Millicell EZ slides (Millipore), followed by iPSCs or ESCs. Cells were washed, fixed in 4% PFA for 10 min, and then permeabilized with 0.1% Triton X-100 for 10 min. For detection of SSEA1, a surface marker of iPSCs/ESCs, the cells were not permeabilized. Nonspecific binding sites on the cells were blocked with blocking buffer (1% BSA in PBS). Cells were stained with antibodies against Oct4, Sox2, Nanog, SSEA1 (1:100) (ab107156, Abcam), HIF2α (1:200) (NB100-122, Novus), me3H3k27 (1:200) (ab6147, Abcam) respectively for 60 min at RT, and then washed with PBS. Then the slides were incubated with goat anti-rabbit antibody conjugated with Alexa Fluor 568 (1:250; Invitrogen) for 1 h. After washing, the nuclear were stained with 4′, 6-diamidino-2-phenylindole (DAPI) (1:1000) (Invitrogen). Cells were observed under confocal microscopy (TCS SP5; Leica, Wetzlar, Germany).

### Teratoma formation and histological analysis

The mice were supplied with sterile water and rodent pellets under the control of the animal facility of the Institution of Cellular and Organismic Biology, Academia Sinica, Taiwan. All procedures were approved by the Institutional Animal Care and Use Committee of Academia Sinica (AS IACUC). Protocol ID: 11-04-166.

The iPSCs (5 × 10^6^) were suspended in DMEM containing 10% FBS, and was injected subcutaneously into the dorsal flank of nude mice. Four weeks after the injection, tumors were surgically dissected from the mice. Samples were fixed in PBS containing 4% PFA, and embedded in paraffin. Sections were stained with hematoxylin and eosin.

### Chimeric mouse production and examination

The mice were supplied with sterile water and rodent pellets under the control of the animal facility of the Institution of Cellular and Organismic Biology, Academia Sinica, Taiwan. All procedures were approved by the Institutional Animal Care and Use Committee of Academia Sinica (AS IACUC). Protocol ID: 11-04-166.

The iPSCs were injected into 3.5 day-old blastocysts by the Transgenic Mouse Model Core Facility of Academia Sinica (Taiwan). For genotyping of the chimera, genomic DNA was extracted using a DNA extraction kit according to the manufacturer’s recommendations (QIAGEN, Valencia, CA, USA). The following primers were used: GFP, forward: 5′-ACGTAAACGGCCACAAGTTC-3′; reverse: 5′-GACTGGGTGCTCAGGTAGTG-3′; GAPDH, forward: 5′-AAGTCGCAGGAGACAACCTG-3′; reverse: 5′-CGTGCAGGACCTCACTCATT-3′. The urogenital ridge region of 13.5 day-old embryos was dissected under anatomical microscopy, and the tissues were mounted. The region was observed under a reflected-light microscope Axio Scope Vario HAL 100 (Carl Zeiss, Inc), and recorded with a ZEISS CoolSNAP camera (Carl Zeiss, Inc) controlled by MetaMorph software (Universal imaging Corporation, PA, USA).

### Bisulfite pyrosequencing

Genomic DNA (1 μg) was isolated using a QIAquick column and be bisulfite converted with a Bisulfite FAST modification kit according to the manufacturer’s instructions (QIAGEN). The promoter regions of mouse *Oct4* were amplified by methylation PCR, and the PCR products were detected by pyrosequencing (Genomics Inc, Taipei, Taiwan). Three groups of biotinylated primers were designed to detect the methylation level at regions on Oct4 promoter. Primers are listed as follows: Assay 1, forward primer: 5′-GGTGGAGGAAGTAGATAATAATGAGAAT-3′; reverse primer (biotinylated): biotin-ACACTTCAAAAACATAATCTCCAAACTC-3′; sequencing primer: 5′-AATAATGAGAATTTTTAGGAGATA-3′. Assay 2, forward primer: 5′-AGAATAGTGTGAGGTGGAGTTTG-3′; reverse primer (biotinylated): biotin-ACCATACTCCAACCACATCCTTCTCTAA-3′; sequencing primer: 5′-AGT TTGGAGATTATGTTTTTG-3′. Assay 3, forward primer: 5′-TAATTAGTTTGG GTTAGAGAAGGATGT-3′; reverse primer (biotinylated): biotin-CCCCCCCTAAAAAAAATATCCCTATAAC-3′; sequencing primer: 5′- GTT AGAGAAGGATGTGG -3′.

### Co-Immunoprecipitation

HEK 293 T cells were co-transfected with HIF2α-V5 and STAT3-Flag expression plasmids using PolyJet DNA Transfection Reagent (SignaGen Laboratories, Ijamsville, MD, USA) and lysed in lysis buffer (50 mM Tris-HCl (pH 7.4), 150 mM NaCl, 1% NP-40) supplemented with protease (Roche). For IP, cell lysates were subjected to incubate with the indicated antibodies overnight at 4 °C. Then, 20 μl of Dynabeads® Protein G (Life Technologies, Inc.) was added and incubated for 2 hr at 4 °C to pull down the antibody-bound protein. The IP samples were washed with lysis buffer five times, denatured in SDS sample buffer, and analyzed by immunoblotting (IB) analysis.

### TACE activity

The ADAM17 activity was measured using the InnoZyme ADAM17 activity kit (Calbiochem). In brief, the cell lysate was harvested and the lysate of each group were loaded into the TACE antibody-coated plated. After 1h-incubation, the lysate was removed and the plate was washed twice. Substrate was added into each well for 5 h at 37 °C. After incubation, the fluorescence of reaction was detected at excitation and emission wavelengths of 324 nm and 405 nm.

### shRNA transduction

For knockdown experiments, mouse EpCAM and luciferase shRNAs were obtained from RNAi core facility (Taipei, Taiwan) (EpCAM shRNA clone #1, 5′-CCTGGTTATATCTACAAGGAA-3′; EpCAM shRNA clone #2, 5′-CTCTGAATGAATATGGTGAAT-3′; Luciferase shRNA clone 5′-CTTCGAAATGTCCGTTCGGTT-3′; HIF2α shRNA clone #1, 5′-CGACAGAATCTTGGAACTGAT-3′; HIF2α shRNA clone #2, 5′-GCCTCATGTCTCCATGTTCAA-3′. The shRNAs were transfected with helper DNA into 293 T cells to generate lentivirus. Cells were selected with 2 μg/mL of puromycin 16 h post-infection.

### Statistical analysis

All data are presented as mean ± SEM for the indicated number of experiments. Unpaired Student’s *t*-test was performed to calculate the statistical significance of the expression percentages versus those of control cultures. A *p*-value of less than 0.05 was considered statistically significant.

## Additional Information

**Accession codes**: The raw and processed RNA-seq data have been deposited in the Gene Expression Omnibus (GEO) database under the accession number GSE71255.

**How to cite this article**: Kuan, I.-I. *et al*. EpEX/EpCAM and Oct4 or Klf4 alone are sufficient to generate induced pluripotent stem cells through STAT3 and HIF2a. *Sci. Rep.* **7**, 41852; doi: 10.1038/srep41852 (2017).

**Publisher's note:** Springer Nature remains neutral with regard to jurisdictional claims in published maps and institutional affiliations.

## Supplementary Material

Supplementary Information

## Figures and Tables

**Table 1:** EpEX and/or EpCAM promote OSKM-mediated reprogramming in Oct4-GFP MEFs on day 25 (n = 9).

Groups	Colony number (50000 cells/well)	Efficiency (%)
OSKM	77.2 ± 6.2	0.15
OSKME	130.5 ± 3.9	0.26
OSKM + EpEX	125.0 ± 14.0	0.25
OSKME + EpEX	178.3 ± 3.6	0.36

**Table 2:** EpCAM and EpEX are sufficient to generate iPSCs with Oct4 or Klf4 in Oct4-GFP MEFs on day 25 (n = 9).

Groups	Colony number (20000 cells/well)	Efficiency (%)
O	0	0
S	0	0
K	0	0
OE	0	0
SE	0	0
KE	0	0
O + EpEX	0	0
S + EpEX	0	0
K + EpEX	0	0
EpEX	0	0
OE + EpEX	9.3 ± 2.0	0.05
SE + EpEX	0	0
KE + EpEX	5.3 ± 1.0	0.03
E + EpEX	0	0
OSKM	36.8 ± 5.0	0.18
