# Supplementary material for: EpEX/EpCAM and Oct4 or Klf4 alone are sufficient to generate induced pluripotent stem cells through STAT3 and HIF2α
Source: Sci Rep. 2017 Feb 3;7:41852. doi: 10.1038/srep41852 (PMC5291097; doi:10.1038/srep41852)
Supplement: Supplementary Information [file srep41852-s1.pdf]

Supplementary information

EpEX/EpCAM and Oct4 or Klf4 alone are sufficient to generate induced pluripotent stem cells through STAT3 and HIF2α

I-I Kuan[1, 2], Kang-Hao Liang[1], Yi-Ping Wang[1, 3], Ting-Wen Kuo[1, 4], Yaa-Jyuhn James Meir[5], Sareina Chiung-Yuan Wu[5], Shang-Chih Yang[6], Jean Lu[6*] and Han-Chung Wu[1, 6*]

[1]Institute of Cellular and Organismic Biology, Academia Sinica, Taipei, Taiwan
[2]Department of Life Science, National Taiwan University, Taipei, Taiwan
[3]School of Dentistry, National Taiwan University, Taipei, Taiwan
[4]Department of Clinical Medicine, School of Medicine, Zhejang University
[5]Institute of Molecular Medicine, College of Medicine, Chang Gung University, Tao-Yuan, Taiwan
[6]Genomics Research Center, Academia Sinica, Taipei, Taiwan

[*]Correspondence should be addressed to H.C.W. (hcw0928@gate.sinica.edu.tw) or Jean Lu (jeanlu@gate.sinica.edu.tw)

Dr. Han-Chung Wu
Institute of Cellular and Organismic Biology, Academia Sinica
128 Academia Road, Section 2, Nankang, Taipei 11529, Taiwan
Tel: 886-2-2789-9515
Fax: 886-2-2785-8059
E-mail: hcw0928@gate.sinica.edu.tw

Dr. Jean Lu
Genomics Research Center, Academia Sinica
128 Academia Road, Section 2, Nankang, Taipei 11529, Taiwan
Tel: 886-2-2789-9580 ext 601
Fax: 886-2-2789-9587
E-mail: jeanlu@gate.sinica.edu.tw

Supplementary Figure S1

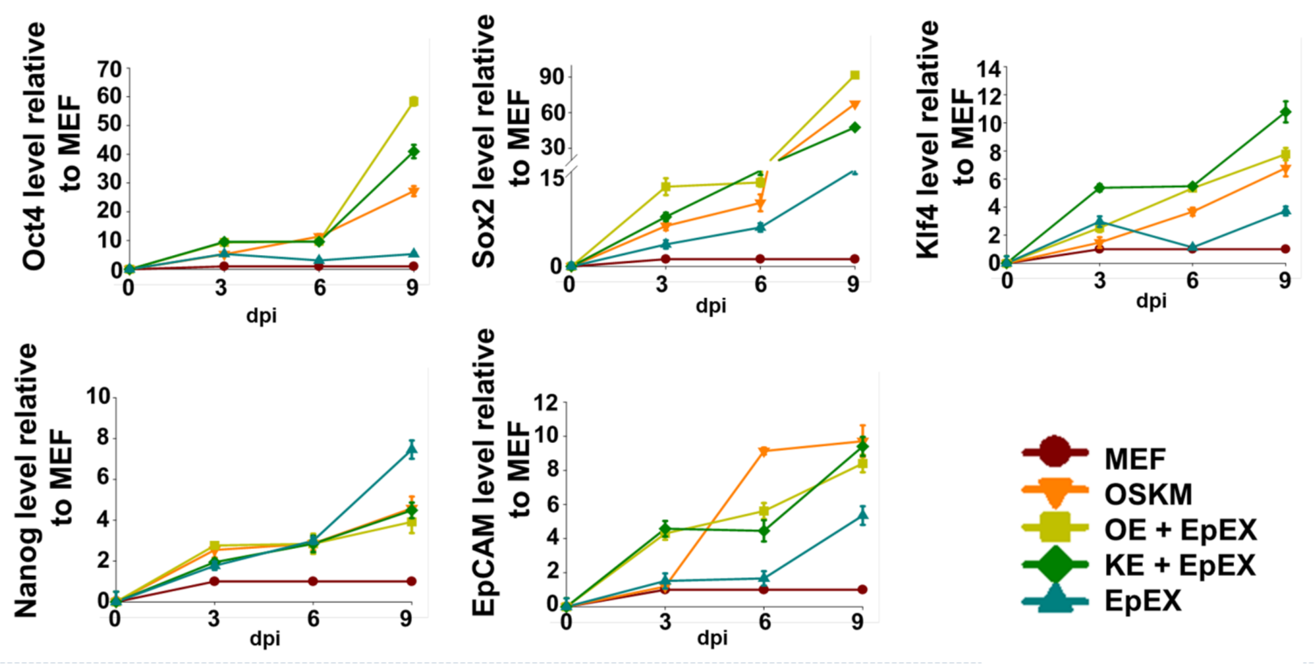

Supplementary figure S1. The kinetic levels of pluripotency factors during reprogramming. MEFs were transfected with OSKM, OE, KE and treated with EpEX, after transfection, cells were stimulated with doxycycline (1.5 μg/mL) and cells were harvested on day 3, 6, 9. Pluripotent factors (Oct4, Sox2, Klf4, Nanog, EpCAM) were detected by Western blotting (n = 3).

Supplementary Figure S2

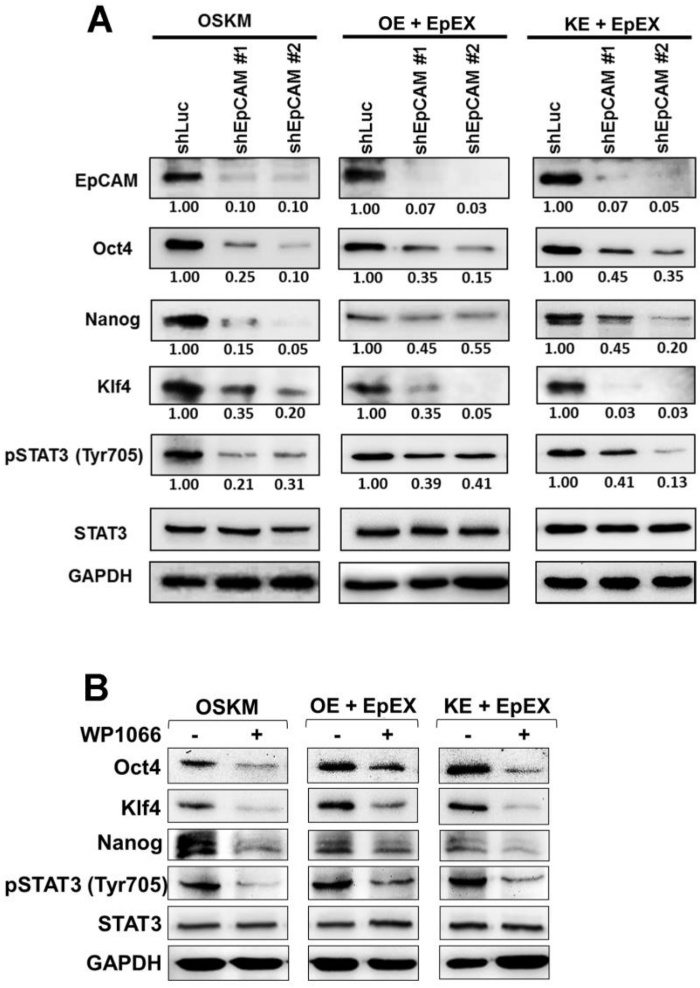

Supplementary figure S2. EpCAM and STAT3 play critical roles in pluripotency maintenance and formation of iPSCs, and iPSCs formation was abolished by inhibition of EpCAM and STAT3. (A) iPSCs were knocked down by EpCAM shRNA (two clones, #1 and #2) and the protein level of EpCAM and pluripotent factors (Oct4, Nanog, Klf4) were detected by Western blotting. In addition, the protein expression of STAT3 and phospho-STAT3 were detected by Western blotting (n = 3). (B) iPSCs were treated by a STAT3 inhibitor, WP1066 (10 µM) for 24 h. Cells were harvested

and the protein level of pluripotent factors (Oct4, Klf4, Nanog) were detected by Western blotting. In addition, the protein expression of STAT3 and phospho-STAT3 were detected by Western blotting (n = 3).

Supplementary Figure S3

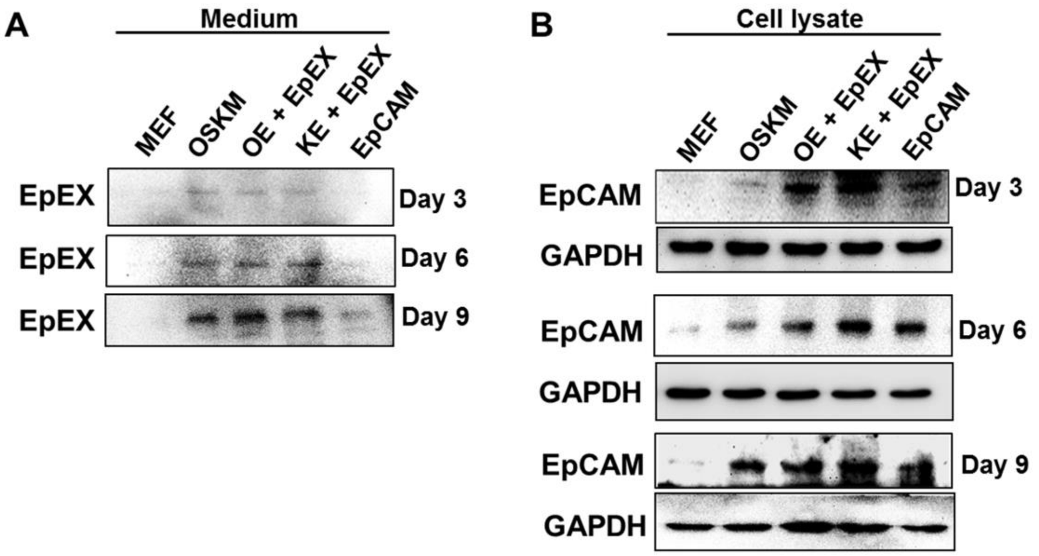

Supplementary figure S3. The expression of EpCAM and EpEX during iPSCs reprogramming at day 3, day 6 and day 9. The expressions of EpCAM and EpEX were detected by Western blotting. (A) The expression of EpEX in conditioned medium was detected by Western blotting. (B) The expression of EpCAM in cell lysate was detected by anti-EpCAM antibody.

Supplementary Figure S4

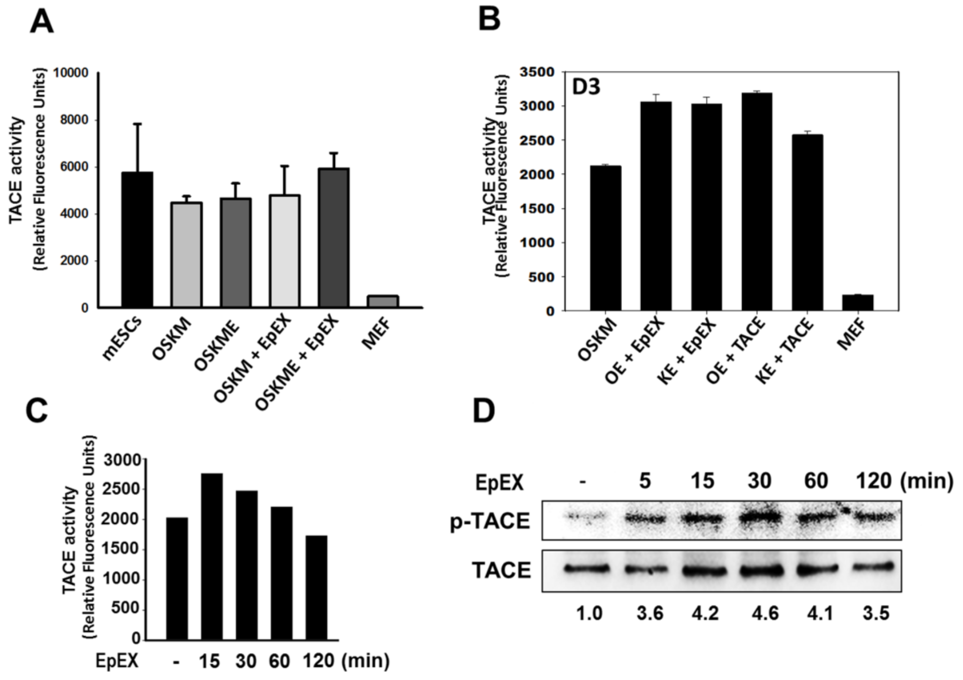

Supplementary figure S4. The phosphorylation of TACE and its activity were induced in MEFs. (A) The activity of TACE in iPSCs and mouse ESCs. The lysate of mESCs and iPSCs was harvested and the activity of TACE was examined. TACE activity is highly elevated in iPSCs and mESCs. (B) The activity of TACE in MEFs. The lysate was harvested and the activity of TACE was examined in each group of early reprogramming induction on day 3. (C) MEFs were stimulated by EpEX at indicated time, and the activity of TACE was detected. (D) Western blot analysis was performed to detect the protein phosphorylation. MEFs were stimulated by EpEX (1 μg/mL) at indicated time. The TACE phosphorylation was detected by antibody against the phosphorylation site Thr735 of TACE.

Supplementary Figure S5

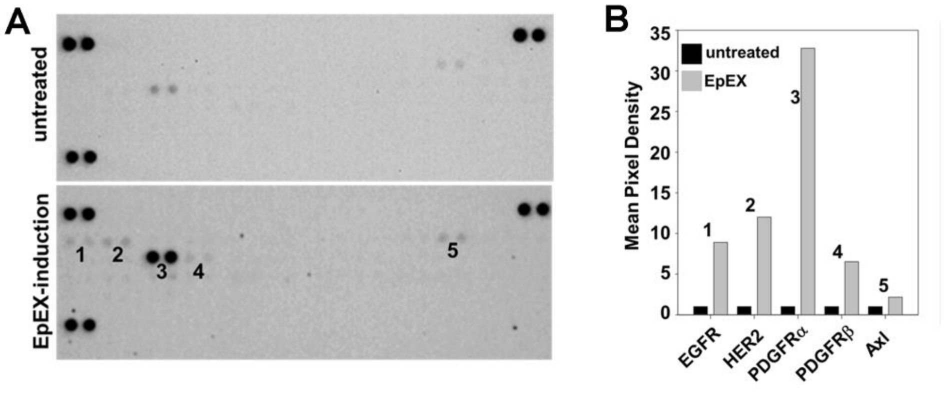

Supplementary figure S5. The effect of EpEX on Receptor Tyrosine Kinase. (A) The phospho-kinase array detects phosphorylated receptor tyrosine kinase in untreated and EpEX-treated MEFs. (B) The quantification by mean pixel density showed that six phosphorylated proteins were regulated by EpEX.

Supplementary Figure S6

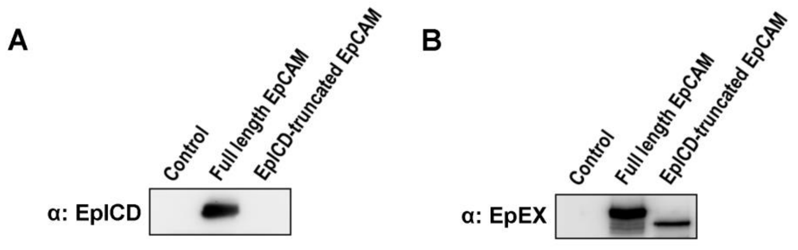

Supplementary figure 6. The expression of EpICD-truncated EpCAM. (A) The expression of EpICD-truncated EpCAM was detected by Western blotting with E144 antibody, which has the recognition site at C-terminal (EpICD). (B) Western blotting with G8.8 antibody, which has recognition site at N-terminal (EpEX).

Supplementary Figure S7

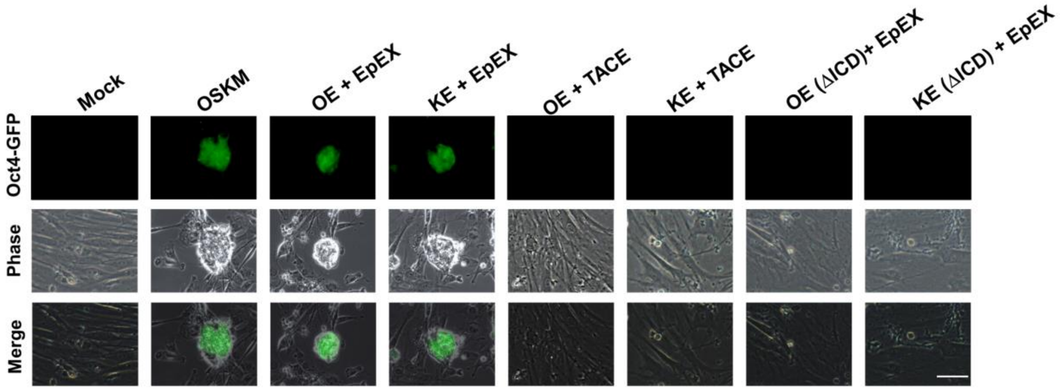

Supplementary figure 7. iPSCs formation on day 20. iPSC morphology was observed at day 20 after induction. Reprogramming of Oct4-GFP MEFs was induced by transfection of OSKM, OE + EpEX, and KE + EpEX with or with TACE, and EpICD-truncated EpCAM with Oct4 or klf4 with EpEX treatment (n = 3). Scale bar: 25 μm.
